# Environmental Flows Can Reduce the Encroachment of Terrestrial Vegetation into River Channels: A Systematic Literature Review

**DOI:** 10.1007/s00267-013-0147-0

**Published:** 2013-08-17

**Authors:** Kimberly A. Miller, J. Angus Webb, Siobhan C. de Little, Michael J. Stewardson

**Affiliations:** 1 Department of Infrastructure Engineering, The University of Melbourne, Parkville, VIC 3010 Australia; 2 School of Resource Management and Geography, The University of Melbourne, Parkville, VIC Australia

**Keywords:** Causal criteria, Eco Evidence, Environmental flows, Riparian, River restoration, Systematic review

## Abstract

Encroachment of riparian vegetation into regulated river channels exerts control over fluvial processes, channel morphology, and aquatic ecology. Reducing encroachment of terrestrial vegetation is an oft-cited objective of environmental flow recommendations, but there has been no systematic assessment of the evidence for and against the widely-accepted cause-and-effect mechanisms involved. We systematically reviewed the literature to test whether environmental flows can reduce the encroachment of terrestrial vegetation into river channels. We quantified the level of support for five explicit cause-effect hypotheses drawn from a conceptual model of the effects of flow on vegetation. We found that greater inundation, variously expressed as changes in the area, depth, duration, frequency, seasonality, and volume of surface water, generally reduces riparian vegetation abundance in channels, but most studies did not investigate the specific mechanisms causing these changes. Those that did show that increased inundation results in increased mortality, but also increased germination. The evidence was insufficient to determine whether increased inundation decreases reproduction. Our results contribute to hydro-ecological understanding by using the published literature to test for general cause-effect relationships between flow regime and terrestrial vegetation encroachment. Reviews of this nature provide robust support for flow management, and are more defensible than expert judgement-based approaches. Overall, we predict that restoration of more natural flow regimes will reduce encroachment of terrestrial vegetation into regulated river channels, partly through increased mortality. Conversely, infrequent deliveries of environmental flows may actually increase germination and subsequent encroachment.

**Electronic supplementary material:**

The online version of this article (doi:10.1007/s00267-013-0147-0) contains supplementary material, which is available to authorized users.

## Introduction

### Encroachment of Terrestrial Vegetation into Regulated River Channels

Regulation of rivers, and the resulting alteration of flow, threatens ecosystem functions and biodiversity globally (Nilsson and others 2005; Dudgeon and others 2006). Among many other effects, river regulation can result in the encroachment of terrestrial vegetation into channels (Erskine and others 1999; Bejarano and others 2011; Bejarano and Sordo-Ward 2011). Moreover, the extent of encroachment can increase with greater reductions in flow (Poff and Zimmerman 2010).

Vegetation encroachment results from a predictable set of conditions, and has well-defined consequences for riverine environments. Frequent high flow events result in regular deposition and removal of sediments from channels. Deposited sediment provides suitable substrate for the germination of terrestrial vegetation, which in turn stabilizes the sediment (Benn and Erskine 1994). When flow is reduced over many years, the vegetation may establish in the base of the channel. The development of large-statured terrestrial vegetation in channels results in a reciprocal relationship with hydrogeomorphic processes (Corenblit and others 2007), with encroachment exerting control over fluvial processes, and in turn, channel morphology, and aquatic ecology (Hickin 1984). Riparian terrestrial vegetation can be responsible for the largest amount of energy loss in fluvial corridors (Nepf and Vivoni 2000). The “clogging” of channels with terrestrial vegetation and subsequent energy losses change aquatic habitat availability and alter aquatic ecosystems.

Riparian and aquatic species are well-adapted to survive and exploit the natural flow regime (Lytle and Poff 2004). Therefore, river restoration often relies on environmental flows designed to reinstate a more natural flow regime (e.g., Rood and others 2005; Konrad and others 2012). Environmental flows are deliberate releases of water to benefit the environment (Poff and others 1997). Environmental flow recommendations often include flows expected to remove and/or prevent the encroachment of terrestrial vegetation in channels (e.g., VEWH and others 2011; Konrad and others 2012). While the ecological relationships that underpin recommendations for terrestrial vegetation encroachment are accepted as fact by many in the research and management community, the evidence for and against them often has not been rigorously tested (Sutherland and others 2004).

In this study, we aimed to rigorously test the assumptions underlying environmental flow recommendations by systematically reviewing the effects of streamflow on riparian vegetation. Our results demonstrate that increased base flows and flooding events of longer duration can prevent the encroachment of terrestrial vegetation into regulated river channels. However, we also show that infrequent inundation may actually increase the germination of terrestrial species, potentially exacerbating encroachment.

### Systematic Reviews to Guide Evidence-Based Environmental Management

Experience-based models of environmental management, such as those described above, have sometimes been proven false after systematic reviews. For example, there is little evidence that in-stream structures improve the production of salmonid fishes (Stewart and others 2006), despite the millions of dollars spent annually for just this purpose.

Effective management and restoration relies on understanding the cause-and-effect relationships that determine how environmental stressors influence ecological responses. However, demonstrating causality in ecology is difficult because of natural variability, lack of replication, the presence of confounding influences, and limits to experimental manipulation. When faced with similar issues in studying the causes of disease, epidemiologists developed “causal criteria” in 1960s. Causal criteria analysis is a method for assessing cause-effect hypotheses in the face of weak experimental evidence, and is widely used in medical research (Weed 1997; Tugwell and Haynes 2006). The approach commonly uses a systematic review to test cause-effect hypotheses. This contrasts to most reviews in ecology, which use a “narrative” approach to survey the current state of knowledge. Systematic reviews of the literature can play a key role in the move toward evidence-based environmental policy and management (Pullin and others 2009). Conceptual models underpin the recommendations to use environmental flows to reduce terrestrial vegetation encroachment. Testing such models against the available scientific evidence strengthens their credibility, may provide new recommendations, and informs the development of statistical models to test the effects of environmental flows.

Our review was conducted using Eco Evidence, a freely available method (Norris and others 2012), with an online database of evidence and supporting software (available from www.toolkit.net.au/tools/eco-evidence, Webb and others 2011). Eco Evidence was recently developed to facilitate systematic review and causal criteria analysis in environmental science, by employing the literature as a source of evidence. The history and logic behind causal criteria generally, and the Eco Evidence framework specifically, are described in detail elsewhere (Hill 1965; Susser 1991; Nichols and others 2011; Norris and others 2012). The Eco Evidence framework relies on the concept of “evidence items,” meaning the atomized findings of studies linking a putative cause (environmental stressor) and effect (ecological response). The framework has several advantages over narrative reviews, including standard terms for classifying causes and effects, standard criteria for evaluating the quality of each study, complete transparency in the review methodology, repeatability of the results, the ability to separately evaluate each linkage in a conceptual model (and thus ask more specific ecological questions), and a more concise and targeted review of the literature (Norris and others 2008; Grove and others 2012). In terms of the analytical effort required, causal criteria analysis using Eco Evidence provides a middle ground between narrative reviews and quantitative meta-analysis, a method more commonly used in systematic reviews. There is no requirement to extract effect size information and convert it to a standard scale for statistical analysis. These features make Eco Evidence particularly relevant to management applications, where government agencies often do not have the time, money or expertise to undertake meta-analyses, but wish to achieve a greater degree of rigor than is possible with a narrative overview of the literature before making management decisions. For example, Norris and Liston (2005) found evidence in the literature that adding artificial habitat structures would benefit Macquarie perch (*Macquaria australasica*). In conjunction with experimental data, water managers expanding the Cotter Reservoir (ACTEW) built extensive infrastructure to protect this endangered species (Lintermans and others 2008).

Moreover, causal criteria analyses may also be more representative of the range of evidence than meta-analyses. Studies that find evidence of an association between a hypothesized cause and effect are more likely to report the summary statistics necessary for meta-analysis, and are therefore overrepresented in these analyses (Bekkering and others 2008). Studies that are inappropriate for meta-analysis, in particular those with negative (i.e., no association) results, can be included in an Eco Evidence analysis. This larger pool of data may reduce the potential for publication bias in the analysis (Greet and others 2011).

## Literature Review Method

**Fig. 1:**
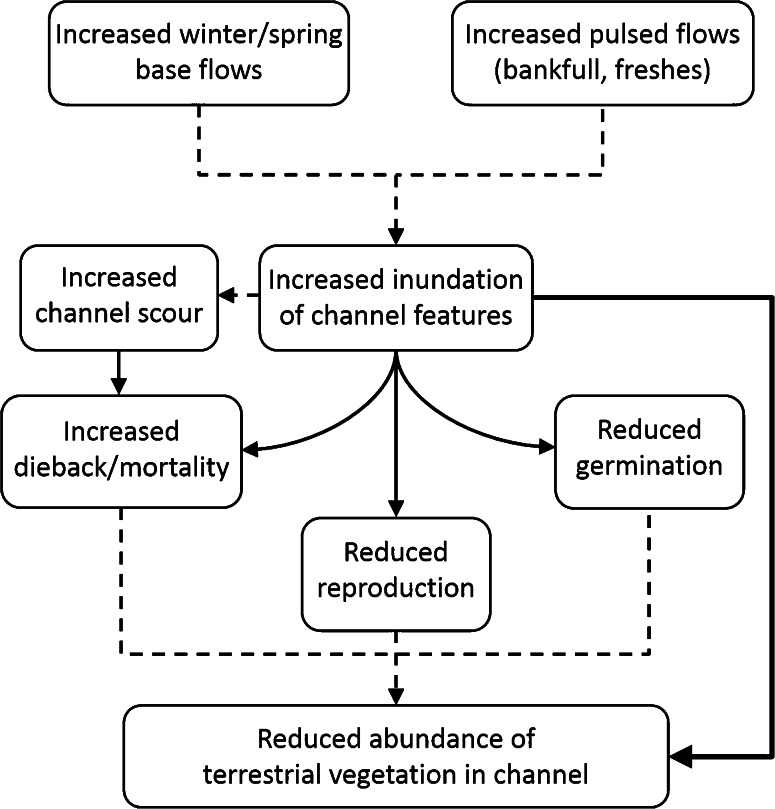
Conceptual model of the relationship between environmental flows and the reduction of encroachment of terrestrial riparian vegetation into channels. Our literature review focused on testing the five cause-effect hypotheses indicated with *solid lines*. *Broken lines* represent assumed links that were not tested. Environmental flows, delivered as either higher baseflows or pulsed flows, lead to greater inundation of the channel because of changes in surface water area, depth, duration, frequency, seasonality, and volume. Inundation itself can reduce germination and reproduction, or increase mortality. An increase in flow volume or velocity may scour the channel, leading to greater mortality through physical removal of vegetation. Collectively, these responses reduce abundance of terrestrial vegetation in channels. The non-specific link between inundation and a reduction in abundance was also evaluated, to include studies that did not identify which of several mechanisms result in the change in abundance

We developed a simple conceptual model of the effects of environmental flows on terrestrial vegetation encroachment in lowland rivers that identifies multiple relevant, testable hypotheses (Fig. 1). The scope of the review was determined by a larger research project that focuses on ecological responses to streamflow in lowland perennial rivers. Our hypothesized conceptual model relates to terrestrial riparian vegetation that germinates, grows, and/or reproduces on dry to saturated or flooded soils, including the plant functional groups of terrestrial-dry, terrestrial-damp, and emergent species (Casanova and Brock 2000). Changes in inundation regimes are variously described in the literature as changes in surface water area, depth, duration, frequency, timing (seasonality), and magnitude (volume) because of the differences in scale and focus of each study (Richter and others 1996). We collectively refer to any of these changes in surface water as “inundation” in this review. The specific hypotheses inherent within the conceptual model were: (i) an increase in sediment scour will cause an increase in plant mortality, (ii) an increase in inundation will cause an increase in mortality, (iii) an increase in inundation will lead to a decrease in reproduction, (iv) an increase in inundation will cause a decrease in seed germination, and (v) an increase in inundation will lead to a decrease in abundance. This last hypothesis recognizes that many studies describe ecological patterns, but do not investigate specific mechanisms. We then conducted a literature search and systematic review using Eco Evidence to test these hypotheses. Briefly, each study was reviewed for relevant evidence items, which were given a weighting based on the study design according to the pre-defined rules in Eco Evidence (Norris and others 2012). After extracting evidence items, we assessed the level of support for each cause-effect hypothesis in the conceptual model and for the overall question. These steps are detailed below.

### Search Strategy and Study Inclusion

We searched the published literature using ISI Web of Science on 15 September 2011. In previous searches on riparian vegetation, this database captured ~88 % of search results of three major databases (Web of Science, SCOPUS, and Expanded Academic ASAP; author’s unpublished data). We conducted separate literature searches for each cause-effect hypothesis identified in the conceptual model (five hypotheses in total; Tables 1, 2). Three limiting term searches narrowed results to studies of flows on riparian vegetation (Table 1). These were combined, as appropriate, with search strings for inundation, scour, and each vegetation response (Tables 1, 2).

**Table 1:** Search terms for limiting term searches (TS), flow descriptors, and vegetation responses

Search	Terms
Limiting TS1	Vegetation OR plant OR “terrestrial-dry” OR “terrestrial-damp”
Limiting TS2	Invas* OR exotic OR terrest*
Limiting TS3	Channel OR river OR stream OR creek OR inchannel
Inundation	Inundat* OR bankfull OR flow$ OR “water regime” OR “water-level” or hydroperio* OR “pulse-flood*” OR “flood release*” OR freshes OR flood$
Scour	Scour*
Mortality	Mortality OR dieback OR surviv* OR death
Reproduction	Reproduc* OR “seed-bank” OR seedbank OR “seed set” OR propagat* OR flower
Germination	Germina* OR seedling OR sapling OR growth
Abundance	Abundan* OR density OR cover OR “population size”

Asterisks are wildcards to represent any group of characters; the dollar sign represents zero or one character

**Table 2:** Complex search operators used to search the published literature

Hypothesis tested		Boolean search operator	Search Hits
Cause (flow)	Effect (vegetation)		
Scour	Mortality	TS1+TS2+TS3+Scour+Mortality	5
Inundation	Mortality	TS1+TS2+TS3+Inundation+Mortality	84
Inundation	Reproduction	TS1+TS2+TS3+Inundation+Reproduction	78
Inundation	Germination	TS1+TS2+TS3+Inundation+Germination	210
Inundation	Abundance	TS1+TS2+TS3+Inundation+Abundance	357

Single cause-effect hypotheses were investigated with combinations of search terms for flow and vegetation responses

We read the titles and abstracts for all studies identified in our literature search. Studies were considered relevant to our review if they presented primary data on the responses of terrestrial vegetation on lowland riverbanks or in channels, to changes in inundation regime. Studies from regulated and unregulated rivers, as well as comparable laboratory experiments were considered relevant. The vegetation response did not have to be the primary focus of the study; for example, the impacts of a scouring flood may have been described in a study comparing sites with differing levels of livestock access. The data could refer to either an increase or decrease in flows, and may be a result of natural variation in flow or anthropogenic streamflow alteration. We categorized relevant papers by the cause-effect hypotheses they informed. Scour was distinguished from inundation as studies that assessed changes in surface water volume or velocity, and indicated physical removal of vegetation.

One cause-effect hypothesis (increase in inundation causes a decrease in abundance) resulted in >100 relevant studies. In the Eco Evidence framework, clear results regarding the support or refutation of a hypothesis can be obtained with a sample from the relevant literature, rather than a complete review (Norris and others 2012). With this in mind, and to efficiently use the resources available for our review, we applied a cut-off of 20 randomly-selected evidence items for this hypothesis. This was approximately double the amount of evidence available for the other hypotheses, and insures that an informative result will be reached during analysis.

### Extraction of Evidence Items

Evidence from each relevant study was extracted according to the standard methodology in Eco Evidence (Nichols and others 2011; Norris and others 2012), and entered into the online Eco Evidence database (Webb and others 2011). We determined the hydrological cause and ecological effect for each evidence item, and recorded the trajectories of both cause and effect (increase, decrease, change, no change; e.g., “increase in flood duration, no change in germination”). The trajectories of the cause and effect determine whether or not an evidence item is consistent with the hypothesized trajectories, and thus whether it supports or refutes the hypothesis.

Following the definitions of Nichols and others (2011), we determined the type of study design from the standard list of categories (“Spatial gradient,” “Temporal gradient,” “Before–After,” “Control–Impact,” “Before–After/Control–Impact (BACI),” or “After-impact-only”) and the number of independent control and impact sampling units. By definition, gradient designs must include at least three sampling points, but may only come from one independent sampling unit (e.g., a gradient of flood magnitude along one river over several years). Eco Evidence uses this information to weight individual evidence items for analysis. These “evidence weights” can range from 1 to 10; studies that better control for confounding variables and/or with greater replication are given a higher weighting, as they are less likely to lead to spurious results (Norris and others 2012). For example, evidence items from a before–after study on one river would receive an evidence weight of 2, whereas those from a BACI study, conducted with one control river and two impacted rivers, would receive an evidence weight of 8. The evidence weights and threshold (described below) were derived from an expert consultation process during the development of the Eco Evidence method (Norris and others 2012). They can be altered prior to undertaking a review if such a change is justified by changing the default settings in the Eco Evidence desktop analysis software (e.g., Grove and others 2012). In this case, we used the default weights, which have proved useful for other reviews of the effects of water regime on vegetation (Greet and others 2011; Webb and others 2012b).

### Data Synthesis

Using the Eco Evidence desktop analysis software, the individual evidence weights that support the hypothesis and those that refute it were summed to evaluate support for each hypothesis in the conceptual model. We used the default threshold of 20 summed points for reaching conclusions. This threshold means that a few high-quality studies are sufficient to support (or refute) a hypothesis, but many weaker studies would be needed to reach the same conclusion (e.g., three studies with a weight of 7 or seven studies with a mean weight of 3).

Four outcomes are possible, based on the number of summed points supporting and refuting the hypothesis. “Support for hypothesis” is achieved when at least 20 summed points lie in favor of the hypothesis, and fewer than 20 points refute it. The hypothesis is falsified by findings of either “Support for Alternate Hypothesis” (at least 20 points refute the hypothesis and fewer than 20 support it) or “Inconsistent evidence” (at least 20 points support and refute the hypothesis). The latter may call for a re-examination the initial conceptual model and/or refining the scope of the hypothesis. “Insufficient evidence” occurs when fewer than 20 points support and refute the hypothesis and no further relevant studies can be found, implying that one cannot reach a conclusion based on the available evidence. These outcomes, like *P* values for significance testing, should not be applied without consideration. For example, if 20 summed points support a hypothesis, and 19 points refute it, a judgement of “Support for hypothesis” is unreasonable, and the evidence should be judged as inconsistent. Conversely, if 150 summed points support the hypothesis, and 20 refute it, a judgement of “Support for hypothesis” may be more reasonable than “Inconsistent evidence” (Harrison 2010).

Lastly, we considered the conclusions for each cause-effect linkage collectively, in order to answer the primary question. An overall finding of support for the primary question does not necessarily require support for each of the cause-effect hypotheses considered (e.g., Greet and others 2011).

## Results

Our searches resulted in 734 hits for 489 unique papers (Table 2). Of these, 29.0 % were deemed relevant to our review after reading the titles and abstracts, a proportion similar to that found in previous studies of responses to flow alteration (Webb and others 2012a). The evidence used in this study is available for re-use from the Eco Evidence database, and can be located by searching the “Question” field for “#Encroachment.” None of the standard terms that describe water regime in the current list of standard terms in the Eco Evidence database sufficiently captured the variation in inundation inherent in our hypotheses. Thus, we defined a new cause term of “Inundation” in the analysis file, and pooled results from studies that are classified in the database as studying changes in surface water area, depth, duration, frequency, seasonality, and volume.

We found support for three of the five cause-effect hypotheses in our conceptual model, support for the alternate hypothesis for the fourth, and insufficient evidence for the fifth (Table 3, Supplementary material S1−5). Overall, the evidence supported the hypothesis that greater inundation reduces riparian vegetation abundance in channels. However, most of these studies did not investigate the specific life-history traits that were affected and caused the reduction in abundance. The average evidence weight per study was 3.7 (range 1–9). The conclusion was based on 1 BACI study, 14 gradient models, 1 before–after study, 3 control–impact studies, and 1 after-impact-only study.

**Table 3:** Results of the Eco Evidence analysis of each cause-effect linkage from our conceptual model

Hypothesis tested		Number of evidence items	Evidence points			References	
Cause (flow)	Effect (vegetation)		Supporting hypothesis	Refuting hypothesis	Conclusion	Supporting hypothesis	Refuting hypothesis
Scour, increase	Mortality, increase	12	28	10	Support for hypothesis	Irvine and West (1979), Auble and others (1997), Stromberg (1997), Friedman and Auble (1999), Acker and others (2003), Pettit and others (2005), Polzin and Rood (2006), Braatne and others (2007), Beche and others (2009), Shafroth and others (2010)	Stromberg and others (1993), Hooke and Mant (2000)
Inundation, increase	Mortality, increase	10	41	7	Support for hypothesis	Stromberg and others (1993), Auble and others (1997), Friedman and Auble (1999), Lesica and Miles (2004), van Eck and others (2004), van Eck and others (2006), Stokes (2008), Mayence and others (2010)	Dawe and Reekie (2007)
Inundation, increase	Reproduction, decrease	5	11	10	Insufficient evidence	Tabacchi and others (2005), Dawe and Reekie (2007)	Taylor and Ganf (2005), Beche and others (2009), Wang and others (2011)
Inundation, increase	Germination, decrease	11	13	24	Support for alternate hypothesis	Cooper and others (2003), Braatne and others (2007), Gurnell and others (2007), Cui and others (2010)	Auble and others (1997), Stromberg (1998), Pettit and others (2001), Burgess and others (2005), Florentine and Westbrooke (2005), Westbrooke and Florentine (2005), Stokes (2008)
Inundation, increase	Abundance, decrease	20	60	13	Support for hypothesis	Irvine and West (1979), Pettit and others (2001), Riis and others (2001), van Eck and others (2004), Florentine and Westbrooke (2005), Taylor and Ganf (2005), van Eck and others (2006), Stromberg and others (2007), Jenkins and others (2008), Whyte and others (2008), Catford and Downes (2010), Cui and others (2010), Toth (2010a, b), Catford and others (2011)	Shafroth and others (1998), Chambers and others (2002), Tiegs and others (2005), Stokes and others (2010), Wang and others (2011)

The summed evidence points that support and refute each hypothesis determine the conclusion

We found support for the two hypothesized mechanisms whereby increased flow can increase vegetation mortality: scour and inundation. The average evidence weight per study on scour was 3.2 (range 2–8). The conclusion was based on 1 BACI study, 5 gradient models, and 6 before–after studies. The conclusion on inundation was based on 1 BACI study, 6 gradient models, 2 before–after studies, and 1 control–impact study. The average evidence weight per study on inundation was 4.8 (range 2–9).

The hypothesis that increased inundation decreases germination was refuted by the evidence, and the alternate hypothesis was supported––i.e., germination is not decreased (Table 3). This conclusion was based on 7 gradient models and 4 control–impact studies. The average evidence weight per study was 3.4 (range 3–4).

The evidence was insufficient to determine whether inundation decreases reproduction, as we found only five relevant studies from 489 studies that were located in the literature search (Table 3). The average evidence weight was 4.2 (range 3–7), and included 2 gradient models and 3 control–impact studies. We conducted a second literature search in an attempt to find further evidence to test this linkage, adding additional search terms identified in the relevant studies. Of 66 hits not identified in the first search, none were deemed relevant to the hypothesis. We therefore concluded that our conclusion of “insufficient evidence” legitimately reflects the amount of evidence available in the literature.

## Discussion

Environmental flows can prevent the encroachment of terrestrial vegetation into lowland river channels when baseflows and pulsed flows mimic the natural flow regime. This finding supports better river management by identifying mechanisms that reduce encroachment, thereby allowing managers to improve riverine ecosystem function, and maintain fluvial processes and channel morphology. Our results also contribute to hydro-ecological understanding by demonstrating generalized cause-effect relationships between flow regime and terrestrial vegetation encroachment, and have identified a knowledge gap regarding the effects of inundation on reproduction.

### Synthesis of Findings

Many studies in our review linked changes in the abundance of terrestrial vegetation with changes in inundation, without identifying the life-history stage(s) affected. These studies provide strong support for our primary question, but cannot inform the mechanistic hypotheses. However, our four mechanistic hypotheses indicate which life-history stages can be targeted by environmental flows to reduce encroachment. The hypotheses that greater flows would increase mortality were both strongly supported by the literature, both through physical removal (scour; e.g., Irvine and West 1979; Polzin and Rood 2006) and flooding stress (e.g., Stokes 2008; Mayence and others 2010). Flooding reduces oxygen availability in soils, and trees may differ in their sensitivity to oxygen deficiency based on evolutionary and environmental factors (Kreuzwieser and others 2004). Environmental flows will most likely be effective in reducing terrestrial vegetation encroachment by increasing mortality when these flows are sufficient to overcome flood-tolerant species (e.g., river red gum, *Eucalyptus camaldulensis*).

Inundating flows that follow the germination period and/or are longer than those frequently reported in the literature should effectively reduce germination. In our review, several studies reported that periodic inundation will actually increase germination rates for riparian species (e.g., Burgess and others 2005; Westbrooke and Florentine 2005). Conversely, above certain thresholds for inundation duration, frequency, volume, and depth, germination rates will decrease (e.g., Gurnell and others 2007). However, a separate review would be required to determine the threshold values. We were only able to find 11 evidence items to assess this hypothesis. These studies included several from riparian zones, where the duration and timing of managed floods would have been designed to stimulate germination on floodplains (e.g., Auble and others 1997; Stromberg 1998). Such studies would have been influential in our overall finding of increased germination with inundation.

We found insufficient evidence to test the hypothesis that increased inundation would decrease reproduction of terrestrial vegetation, and are confident that this represents a true knowledge gap. Two studies provided evidence of decreased sexual reproduction through inundation (Tabacchi and others 2005; Dawe and Reekie 2007), but three studies provided evidence of increased vegetative propagation after large flood events (Taylor and Ganf 2005; Francis 2007; Wang and others 2011). Environmental flows may have very different implications for these two fundamentally-different modes of reproduction, even within a single species (e.g., Barsoum 2001). Further research on each mode of reproduction is needed to reach clear conclusions concerning their sensitivity to inundation.

An important caveat on these conclusions is that our review tests the effects of streamflow on vegetation and therefore identifies how environmental flows can be used to *prevent* vegetation encroachment; our results do not provide evidence that environmental flows can *remove* terrestrial vegetation from channels. Many environmental flow recommendations target the removal of existing terrestrial vegetation from channels in highly-regulated river systems (e.g., EarthTech 2003). Greater inundation alone may reduce encroachment of herbaceous species, shrubs, and small saplings (Stromberg and others 1993; Stromberg 1997; Hooke and Mant 2000; Mayence and others 2010), but may better serve as a complement to manual removal for established terrestrial vegetation, particularly adult trees (e.g., Stromberg and others 1993; Hooke and Mant 2000). Careful management of inundation regimes could then be a primary strategy to prevent re-encroachment.

Lastly, our review excluded evidence from upland and intermittent streams. Differences in the energetics, floristic assemblages, and inundation histories of such streams may require a different conceptual model on the relationships between flow and vegetation responses. Our results should not be extrapolated to such systems.

### Eco Evidence Approach to Systematic Review

Eco Evidence is a relatively novel framework for systematic reviews, and we highlight three key features here of particular relevance to management applications. First, Eco Evidence allows the use of evidence from lower-quality studies to contribute to the overall conclusion. Low-quality studies are very common in environmental science, but are more likely to be confounded by uncontrolled environmental variables than higher-quality studies (Norris and others 2012), and therefore individual low-quality studies may find spurious correlations. However, independent studies conducted in different times, places, and circumstances (e.g., different experimental designs) will be confounded in different ways. Therefore, if a group of individually weak pieces of evidence consistently show the same relationship between a hypothesized cause and effect (i.e., a conclusion of “support for hypothesis), it is unlikely to be a spurious conclusion (US Department of Health and Human Services 2004). A diverse collection of individually weak pieces of evidence can result in a strong conclusion, and actually allows for the inclusion of more of the literature, making the most of the scant evidence available (Norris and others 2012). The mean and range of evidence weights in an analysis provide an indication of how many low- and high-quality studies were used to reach the conclusion. In this review, all conclusions were based on a range of study types with different weaknesses in their design, increasing confidence in the conclusions.

Second, Eco Evidence can allow the reviewer to reach a conclusion using only a sample of the literature rather than an exhaustive review. Fundamentally, this approach rests on the assumption common to all research that a random selection of the possible data should be representative of the entire population––in this case, the available knowledge. The extraction of evidence items requires a careful, systematic dissection of each study (~1–1.5 h/study). Thus, an exhaustive review using the Eco Evidence approach may be prohibitive for generalized hypotheses (Webb and others 2012a). In this review, the specificity of our hypotheses resulted in the exhaustive review of all search hits for the four mechanistic hypotheses, but not the general hypothesis. Reviewing a very large number of relevant studies for a single secondary question would consume extensive resources for a diminishing return. Applying the cut-off of 20 evidence items allowed us to comprehensively address this hypothesis, but not needlessly expend effort conducting an exhaustive review. This consideration is important for management applications, where a manager might have limited resources available for a review. The Eco Evidence approach may be a more probative investigation of the literature, capable of reaching stronger conclusions, than a narrative or other type of quantitative review. Indeed, a recent comparison of an Eco Evidence review to an influential semi-quantitative review (Poff and Zimmerman 2010) found that Eco Evidence reached stronger and more detailed conclusions (Webb and others 2013).

Lastly, Eco Evidence provides complete transparency of the review process. The software produces a standard report that details all evidence used in the assessment, whether it supported or refuted individual hypotheses, and the weightings assigned to individual studies. Because there is no need for statistical inference (unlike meta-analysis), the conclusions can be readily critiqued by experts and non-experts alike.

## Conclusions

The encroachment of terrestrial vegetation into river channels negatively impacts upon fluvial processes, channel morphology, and aquatic ecology. Effective prevention of terrestrial vegetation encroachment is essential for river management and restoration. Systematic reviews provide robust support for environmental management decisions that is more defensible than expert judgement-based approaches. Our review has shown that the restoration of more natural flow regimes that inundate channel features should prevent the encroachment of terrestrial vegetation into river channels. Greater inundation could be achieved through increased base flows and pulsed flows. The reduction of encroachment will mostly result from increased mortality. However, infrequent delivery of pulsed environmental flows may actually increase germination of terrestrial vegetation, and subsequent encroachment. The effects of flow on reproduction are not well-understood, and dedicated research and monitoring of this relationship would improve knowledge for river management. Lastly, while environmental flows may be successful for preventing encroachment of terrestrial vegetation into regulated river channels, they may not be appropriate as the sole strategy for the removal of adult woody vegetation.

## Electronic supplementary material

Below is the link to the electronic supplementary material.

Supplementary material 1 (PDF 150 kb)

Supplementary material 2 (PDF 145 kb)

Supplementary material 3 (PDF 136 kb)

Supplementary material 4 (PDF 148 kb)

Supplementary material 5 (PDF 159 kb)
