# Supplementary material for: Environmental Flows Can Reduce the Encroachment of Terrestrial Vegetation into River Channels: A Systematic Literature Review
Source: Environ Manage. 2013 Aug 17;52(5):1202–12. doi: 10.1007/s00267-013-0147-0 (PMC3825610; doi:10.1007/s00267-013-0147-0)
Supplement: Supplementary file 1 — Supplementary material 1 (PDF 150 kb) [file 267_2013_147_MOESM1_ESM.pdf]

Eco Evidence: Analysis report

Problem

Environmental flows can reduce the encroachment of terrestrial vegetation into river channels: a systematic literature review

Question

An increase in sediment scour will cause an increase in plant mortality.

Context

Studies were considered relevant to our review if they presented primary data on the responses of terrestrial vegetation on lowland riverbanks or in channels, to changes in inundation regime. Studies from regulated and unregulated rivers, as well as comparable laboratory experiments were considered relevant. The vegetation response did not have to be the primary focus of the study; for example, the impacts of a scouring flood may have been described in a study comparing sites with differing levels of livestock access. The data could refer to either an increase or decrease in flows, and may be a result of natural variation in flow or anthropogenic streamflow alteration.

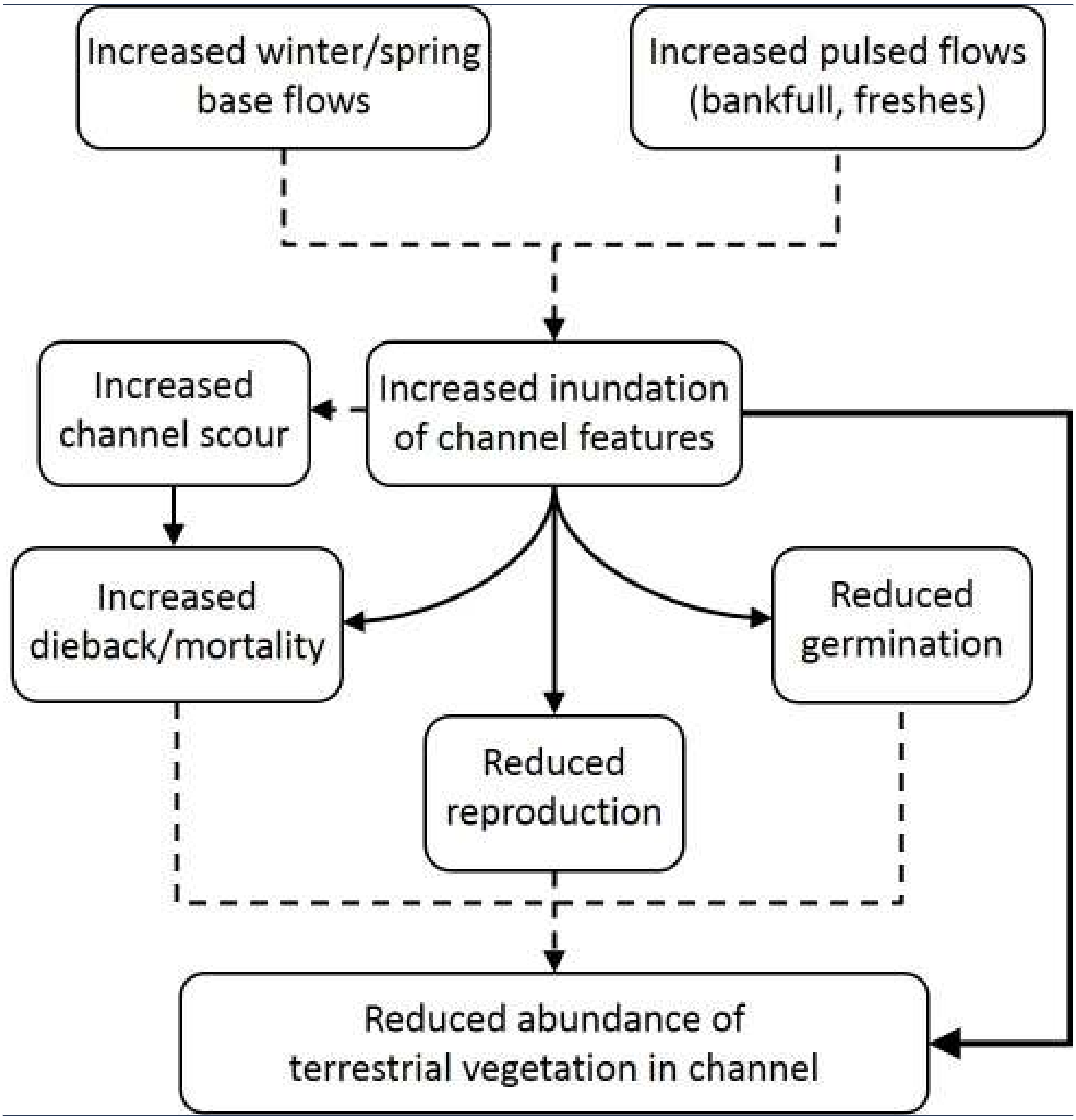

Literature review

Table 1: Results

The evidence according to the 3 major causal criteria shows whether the analysis provides enough support for a causal relationship between the hypothesised effect-cause linkages or alternatively whether there is no support, insufficient evidence or inconsistent evidence for the causal relationship. The minimum requirement for demonstration of a causal relationship is either "Response" or "Dose-response" to be HIGH, and also "Consistency" needs to be HIGH. Also shown are the number of studies and citations contributing to the analysis of each linkage.

Linkage	Conclusion regarding the level of support for the hypothesised linkage	Level of support for each criterion (sum of weights) *			Item counts		Number of studies reporting signs of causal agent in the biota
		Response	Dose-response	Consistency	Evidence items **	Citations	
↑ Scour → ↑ vegetation (mortality)	Support for hypothesis	High (28)	No evidence (0)	High (10)	12	12	6
	Total number of evidence items and citations contributing to causal analysis				12	12	6

* Summed study weights for the different causal criteria. For "Response" and "Dose-response" criteria, if the summed study weight is less than 20 then the level of support is LOW, otherwise it is HIGH. For "Consistency" criteria, if the summed study weight is less than 20 then the level of support is HIGH, otherwise it is LOW.
** The number of relevant evidence items contributing to the analysis. Relevance is determined (and documented) by the user. For evidence to be included, the study must also conduct an appropriate analysis/interpretation. The project file contains the justification for including or excluding each evidence item.

Appendix

Table 2: Evidence relating to each cause-effect linkage

↑ Scour → ↑ vegetation (mortality)

Cause (and trajectory)		Effect (and trajectory)		Supports linkage?	Study details	Weight	Citation
High spring flow (11.9 m^3/s) in one year of the study removed individuals.	Increase	Mortality, total removal, and no subsequent establishment of Russian-olive and honey locust individuals (Elaegnus angustifolia and Gleditsia triacanthos, both terrestrial dry species) within the channel	Increase	Yes	Gradient response model<br>1 (independent)	3	Auble G. T., Scott M. L., Friedman J. M., Back J. and Lee V. J. (1997)
Flood event with peak flows of various magnitude (0-107 cms)	Increase	Little scour. Most herbs were removed by flow, and some shrubs removed by scour at sites with the highest flow. Some mortality of olive and popular trees in the floodplain above channels and flattening of vegetation within channels. Sedimentation in channels and floodplains.	No change	No	BACI or BARI MBACI or Beyond MBACI<br>1 (control);<br>2 (impacted)	8	Hooke, J. M. Mant, J. M. (2000)
Scouring force of high flows	Increase	Scour of invasive Tamarix, as evidence by presence of plants only or predominantly behind large boulders, which shields plants from sheer forces.	Increase	Yes	Gradient response model<br>1 (independent)	3	Irvine, J.R. West, N.E. (1979)
Large flood (6000-7450 cms, 100-y return interval), which exceeds the mean annual flood (289 +/- 221 cms) by and order of magnitude.	Increase	Scour of large sections of riparian plant communities and the deposition of large woody debris piles.	Increase	Yes	Before v. after (no reference/control)<br>0 (control);<br>1 (impacted)	2	Pettit, N. E. Naiman, R. J. Rogers, K. H. Little, J. E. (2005)
Large flood (1-in-100 year recurrence interval) as a result of widespread, intense rain.	Increase	Abrupt scouring of vegetation along the channel	Increase	Yes	Before v. after (no reference/control)<br>0 (control);<br>1 (impacted)	2	Polzin, M. L. Rood, S. B. (2006)
Large winter peak floods (566 cms; 15-20 y recurrence interval), which deposited sediment on a large portion of the floodplain	Increase	High (96-100%) mortality of 2 year-old seedling cohorts of 3 species (Fremont cottonwood, Populus fremontii; Goodding willow, Salix gooddingii; and introduced salt cedar, Tamarix chinensis).	Increase	Yes	Before v. after (no reference/control)<br>0 (control);<br>3 (impacted)	5	Stromberg, J. C. (1997)
Spring pulse floods (experimentally delivered from a dam), with a 1.5-1.7y recurrence interval.	Increase	Mortality (i.e.: reduced stem density) on vegetated bars in channel. Scour and burial by sediment caused higher mortality in smaller, exotic Tamarix seedlings than native Salix seedlings, which were taller and of greater diameter.	Increase	Yes	Before v. after (no reference/control)<br>0 (control);<br>1 (impacted)	2	Shafroth, P. B. Wilcox, A. C. Lytle, D. A. Hickey, J. T. Andersen, D. C. Beauchamp, V. B. Hautzinger, A. McMullen, L. E. Warner, A. (2010)
Peak flow velocity and tractive shear stress (scour) imposed by a 10y return flood (>3000 times base flow) on	Increase	No relationship with mortality of shrubs, saplings, and pole trees of Populus and Salix. Mortality was related to higher water depth, floodplain elevation, and distance to primary channel [not	No change	No	Before v. after (no reference/control)<br>0 (control);<br>1 (impacted)	2	Stromberg J. C., Richter B. D., Patten D. T. and Wolden L. G. (1993)

floodplain vegetation.		included in this evidence item].					
High peak flows that exceed the critical sheer stress required to mobilize sediment	Increase	Scour of box elders (Acer negundo) within the channel due to sediment mobilization (ie: exceeding critical sheer stress). Survival in plots exceeding critical sheer stress was 8%, compared to 19% in plots where critical sheer stress was not exceeded.	Increase	Yes	Before v. after (no reference/control) 0 (control); 1 (impacted)	2	Friedman J. M. and Auble G. T. (1999)
Magnitude of annual peak flows ranging from 28-227 cms over 7 years of study	Increase	High mortality (up to 25% of woody vegetation in years of highest floods in the zone adjacent to the channel ("active floodplain"). Mortality also high in the "quiet floodplain" (adjacent to the active floodplain) during large floods.	Increase	Yes	Gradient response model 1 (independent)	3	Acker, S. A. Gregory, S. Lienkaemper, G. McKee, W. A. Swanson, F. J. Miller, S. D. (2003)
High flows in late summer through winter, in the first two years after recruitment of seedlings	Increase	Mortality of cottonwood seedlings (Populus trichocarpa, terrestrial damp species) due to lethal scouring in the channel	Increase	Yes	Gradient response model 1 (independent)	3	Braatne, J. H. Jamieson, R. Gill, K. M. Rood, S. B. (2007)
Peak river flows in spring, capable of uprooting trees.	Increase	Uprooting (scour) of large trees up to 15 m results in mortality of 44-64% of Populus, Salix, and Alnus trees. The remainder are deposited and continue growth/sprouting.	Increase	Yes	Gradient response model 1 (independent)	3	Francis, R. A. (2007)

Citations

Acker, S. A. Gregory, S. Lienkaemper, G. McKee, W. A. Swanson, F. J. Miller, S. D. (2003) *Composition, complexity, and tree mortality in riparian forests in the central Western Cascades of Oregon*. Forest Ecology and Management

Auble G. T., Scott M. L., Friedman J. M., Back J. and Lee V. J. (1997) *Constraints on establishment of plains cottonwood in an urban riparian preserve*. Wetlands

Braatne, J. H. Jamieson, R. Gill, K. M. Rood, S. B. (2007) *Instream flows and the decline of riparian cottonwoods along the Yakima River, Washington, USA*. River Research and Applications

Francis, R. A. (2007) *Size and position matter: riparian plant establishment from fluvially deposited trees*. Earth Surface Processes and Landforms

Friedman J. M. and Auble G. T. (1999) *Mortality of riparian box elder from sediment mobilization and extended inundation*. Regulated Rivers-Research & Management

Hooke, J. M. Mant, J. M. (2000) *Geomorphological impacts of a flood event on ephemeral channels in SE Spain*. Geomorphology

Irvine, J.R. West, N.E. (1979) *Riparian Tree Species Distribution and Succession along the Lower Escalante River, Utah*. The Southwestern Naturalist

Pettit, N. E. Naiman, R. J. Rogers, K. H. Little, J. E. (2005) *Post-flooding distribution and characteristics of large woody debris piles along the semi-arid Sabie River, South Africa*. River Research and Applications

Polzin, M. L. Rood, S. B. (2006) *Effective disturbance: Seedling safe sites and patch recruitment of riparian cottonwoods after a major flood of a mountain river*. Wetlands

Shafroth, P. B. Wilcox, A. C. Lytle, D. A. Hickey, J. T. Andersen, D. C. Beauchamp, V. B. Hautzinger, A. McMullen, L. E. Warner, A. (2010) *Ecosystem effects of environmental flows: modelling and experimental floods in a dryland river*. Freshwater Biology

Stromberg J. C., Richter B. D., Patten D. T. and Wolden L. G. (1993) *Response of a Sonoran Riparian Forest to a 10-Year Return Flood*. Great Basin Naturalist

Stromberg, J. C. (1997) *Growth and survivorship of Fremont cottonwood, Goodding willow, and salt cedar seedlings after large floods in central Arizona*. Great Basin Naturalist

Table 3. Weights applied in this analysis

Study design type	Weight
BACI or BARI MBACI or Beyond MBACI	4
Gradient response model	3
Before v. after (no reference/control)	2
Reference/control vs. impact (no before)	2
After impact only	1
**Number of independent control locations**	**Weight**
No control locations	0
One control location	2
More than one control location	3
**Number of independent impact locations**	**Weight**
One impacted location	0
Two impacted locations	2
More than two impacted locations	3

Number of locations for gradient response model	Weight
3 independent locations	0
4 independent locations	2
5 independent locations	4
More than 5 independent locations	6
