# Supplementary material for: Environmental Flows Can Reduce the Encroachment of Terrestrial Vegetation into River Channels: A Systematic Literature Review
Source: Environ Manage. 2013 Aug 17;52(5):1202–12. doi: 10.1007/s00267-013-0147-0 (PMC3825610; doi:10.1007/s00267-013-0147-0)
Supplement: Supplementary file 2 — Supplementary material 2 (PDF 145 kb) [file 267_2013_147_MOESM2_ESM.pdf]

Eco Evidence: Analysis report

Problem

Environmental flows can reduce the encroachment of terrestrial vegetation into river channels: a systematic literature review

Question

An increase in inundation will cause an increase in mortality.

Context

Studies were considered relevant to our review if they presented primary data on the responses of terrestrial vegetation on lowland riverbanks or in channels, to changes in inundation regime. Studies from regulated and unregulated rivers, as well as comparable laboratory experiments were considered relevant. The vegetation response did not have to be the primary focus of the study; for example, the impacts of a scouring flood may have been described in a study comparing sites with differing levels of livestock access. The data could refer to either an increase or decrease in flows, and may be a result of natural variation in flow or anthropogenic streamflow alteration.

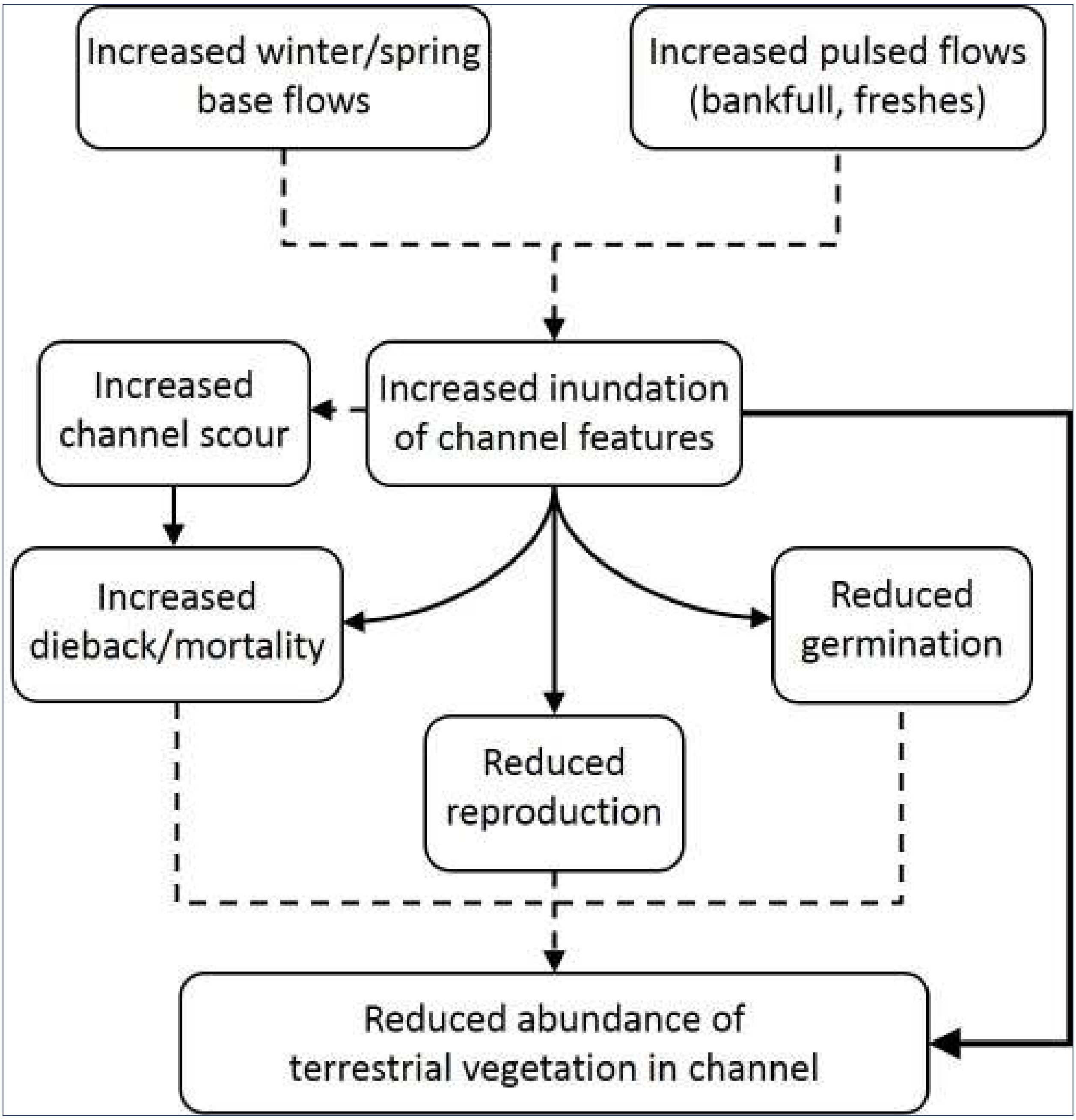

Literature review

Table 1: Results

The evidence according to the 3 major causal criteria shows whether the analysis provides enough support for a causal relationship between the hypothesised effect-cause linkages or alternatively whether there is no support, insufficient evidence or inconsistent evidence for the causal relationship. The minimum requirement for demonstration of a causal relationship is either "Response" or "Dose-response" to be HIGH, and also "Consistency" needs to be HIGH. Also shown are the number of studies and citations contributing to the analysis of each linkage.

Linkage	Conclusion regarding the level of support for the hypothesised linkage	Level of support for each criterion (sum of weights) *			Item counts		Number of studies reporting signs of causal agent in the biota
		Response	Dose-response	Consistency	Evidence items **	Citations	
↑ Inundation → ↑ vegetation (mortality)	Support for hypothesis	High (41)	No evidence (0)	High (7)	10	9	3
	Total number of evidence items and citations contributing to causal analysis				10	9	3

* Summed study weights for the different causal criteria. For "Response" and "Dose-response" criteria, if the summed study weight is less than 20 then the level of support is LOW, otherwise it is HIGH. For "Consistency" criteria, if the summed study weight is less than 20 then the level of support is HIGH, otherwise it is LOW.
** The number of relevant evidence items contributing to the analysis. Relevance is determined (and documented) by the user. For evidence to be included, the study must also conduct an appropriate analysis/interpretation. The project file contains the justification for including or excluding each evidence item.

Appendix

Table 2: Evidence relating to each cause-effect linkage

↑ Inundation → ↑ vegetation (mortality)

Cause (and trajectory)		Effect (and trajectory)		Supports linkage?	Study details	Weight	Citation
High spring flow (11.9 m3/s) in one year of the study.	Increase	Mortality, total removal, and no subsequent establishment of Russian-olive and honey locust individuals (Elaegnus angustifolia and Gleditsia triacanthos, both terrestrial dry species) within the channel.	Increase	Yes	Gradient response model 1 (independent)	3	Auble G. T., Scott M. L., Friedman J. M., Back J. and Lee V. J. (1997)
Complete inundation to 25 cm above the tallest seedling shoots for 42 days.	Increase	High mortality (60%) and inundation stress (40%) of seedlings of the terrestrial damp species, Juncus ingens, when compared to partial inundation, saturated, or moist conditions for the same duration. Authors also found lower shoot, root, and rhizome biomass and rhizome length under complete inundation conditions. [Not included in this evidence item]	Increase	Yes	BACI or BARI MBACI or Beyond MBACI 3 (control); 1 (impacted)	7	Mayence, C. E. Marshall, D. J. Godfree, R. C. (2010)
Magnitude of peak spring flood: 2y return flood (68 cms), 5y return flood (193 cms), and 10y return flood (368 cms)	Increase	Increased mortality of Populus and Salix saplings with maximum annual flood volume.	Increase	Yes	Gradient response model 1 (independent)	3	Stromberg J. C., Richter B. D., Patten D. T. and Wolden L. G. (1993)
Height of water on a floodplain, after a 10y return flood (>3000 times base flow).	Increase	Increased mortality of shrubs, saplings, and pole trees with higher water depth, floodplain elevation, and distance to primary channel.	Increase	Yes	Before v. after (no reference/control) 0 (control); 1 (impacted)	2	Stromberg J. C., Richter B. D., Patten D. T. and Wolden L. G. (1993)
Inundation of plots for more than 85 days during the growing season. Discharge exceeding 114 cms required to inundate plots	Increase	High mortality (91%) of box elder trees (Acer negundo) in plots inundated for 85 days or longer. Mortality was low (8%) when inundation was less than 85 days.	Increase	Yes	Before v. after (no reference/control) 0 (control); 1 (impacted)	2	Friedman J. M. and Auble G. T. (1999)
Hydrocoytle umbellata seedlings, experimental treatments: increase in height of water from 0 cm to 15 cm to 30 cm, or flooded early, mid or late summer for around 35 days.	Change	The proportion of total Hydrocoytle umbellata mass composed of dead tissue (laminas and petioles) at the final harvest did not vary among treatments with the exception that plants flooded late in the growing season had a higher proportion of dead tissue (Table 1). It should be noted that at earlier harvests, the proportion of total mass in dead tissue increased immediately after plants were subjected to flooding (data not presented). This was true regardless of when plants were flooded.	Increase	No	Reference/control vs. impact (no before) 1 (control); 5 (impacted)	7	Dawe, Christine E., Reekie, Edward G. (2007)
Inundation of the floodplain for at least 3 months	Increase	Mortality of invasive tamarisk (Tamarix ramosissima).	Increase	Yes	Gradient response model 1 (independent)	3	Lesica, P. Miles, S. (2004)
Inundation of reservoir	Increase	Mortality of willow seedlings	Increase	Yes	Gradient response model	3	Stokes,

margins					1 (independent)		KE (2008)
Duration of summer and winter flooding in experimental treatments	Increase	Decreased survival of grassland species with longer flood duration. All species survived longer under winter floods than summer floods.	Increase	Yes	Gradient response model 10 (independent)	9	van Eck, Whjm Lenssen, J. P. M. van de Steeg, H. M. Blom, Cwpm de Kroon, H. (2006)
Duration of complete inundation in experimental treatments.	Increase	Mortality of 20 terrestrial plant species increased with longer inundation, but effect was stronger in some species.	Increase	Yes	Gradient response model 20 (independent)	9	van Eck, Whjm van de Steeg, H. M. Blom, Cwpm de Kroon, H. (2004)

Citations

Auble G. T., Scott M. L., Friedman J. M., Back J. and Lee V. J. (1997) *Constraints on establishment of plains cottonwood in an urban riparian preserve*. Wetlands

Dawe, Christine E., Reekie, Edward G. (2007) *The effects of flooding regime on the rare Atlantic coastal plain species Hydrocoytle umbellata*. Canadian Journal of Botany-Revue Canadienne De Botanique

Friedman J. M. and Auble G. T. (1999) *Mortality of riparian box elder from sediment mobilization and extended inundation*. Regulated Rivers-Research & Management

Lesica, P. Miles, S. (2004) *Ecological strategies for managing tamarisk on the CM Russell National Wildlife Refuge, Montana, USA*. Biological Conservation

Mayence, C. E. Marshall, D. J. Godfree, R. C. (2010) *Hydrologic and mechanical control for an invasive wetland plant, Juncus ingens, and implications for rehabilitating and managing Murray River floodplain wetlands, Australia*. Wetlands Ecology and Management

Stokes, KE (2008) *Exotic invasive black willow (Salix nigra) in Australia: influence of hydrological regimes on population dynamics*. PLANT ECOLOGY

Stromberg J. C., Richter B. D., Patten D. T. and Wolden L. G. (1993) *Response of a Sonoran Riparian Forest to a 10-Year Return Flood*. Great Basin Naturalist

van Eck, Whjm Lenssen, J. P. M. van de Steeg, H. M. Blom, Cwpm de Kroon, H. (2006) *Seasonal dependent effects of flooding on plant species survival and zonation: a comparative study of 10 terrestrial grassland species*. Hydrobiologia

van Eck, Whjm van de Steeg, H. M. Blom, Cwpm de Kroon, H. (2004) *Is tolerance to summer flooding correlated with distribution patterns in river floodplains? A comparative study of 20 terrestrial grassland species*. Oikos

Table 3. Weights applied in this analysis

Study design type	Weight
BACI or BARI MBACI or Beyond MBACI	4
Gradient response model	3
Before v. after (no reference/control)	2
Reference/control vs. impact (no before)	2
After impact only	1
**Number of independent control locations**	**Weight**
No control locations	0
One control location	2
More than one control location	3
**Number of independent impact locations**	**Weight**
One impacted location	0
Two impacted locations	2
More than two impacted locations	3
**Number of locations for gradient response model**	**Weight**
3 independent locations	0
4 independent locations	2
5 independent locations	4
More than 5 independent locations	6
