# Supplementary material for: Environmental Flows Can Reduce the Encroachment of Terrestrial Vegetation into River Channels: A Systematic Literature Review
Source: Environ Manage. 2013 Aug 17;52(5):1202–12. doi: 10.1007/s00267-013-0147-0 (PMC3825610; doi:10.1007/s00267-013-0147-0)
Supplement: Supplementary file 3 — Supplementary material 3 (PDF 136 kb) [file 267_2013_147_MOESM3_ESM.pdf]

Eco Evidence: Analysis report

Problem

Environmental flows can reduce the encroachment of terrestrial vegetation into river channels: a systematic literature review

Question

An increase in inundation will lead to a decrease in reproduction.

Context

Studies were considered relevant to our review if they presented primary data on the responses of terrestrial vegetation on lowland riverbanks or in channels, to changes in inundation regime. Studies from regulated and unregulated rivers, as well as comparable laboratory experiments were considered relevant. The vegetation response did not have to be the primary focus of the study; for example, the impacts of a scouring flood may have been described in a study comparing sites with differing levels of livestock access. The data could refer to either an increase or decrease in flows, and may be a result of natural variation in flow or anthropogenic streamflow alteration.

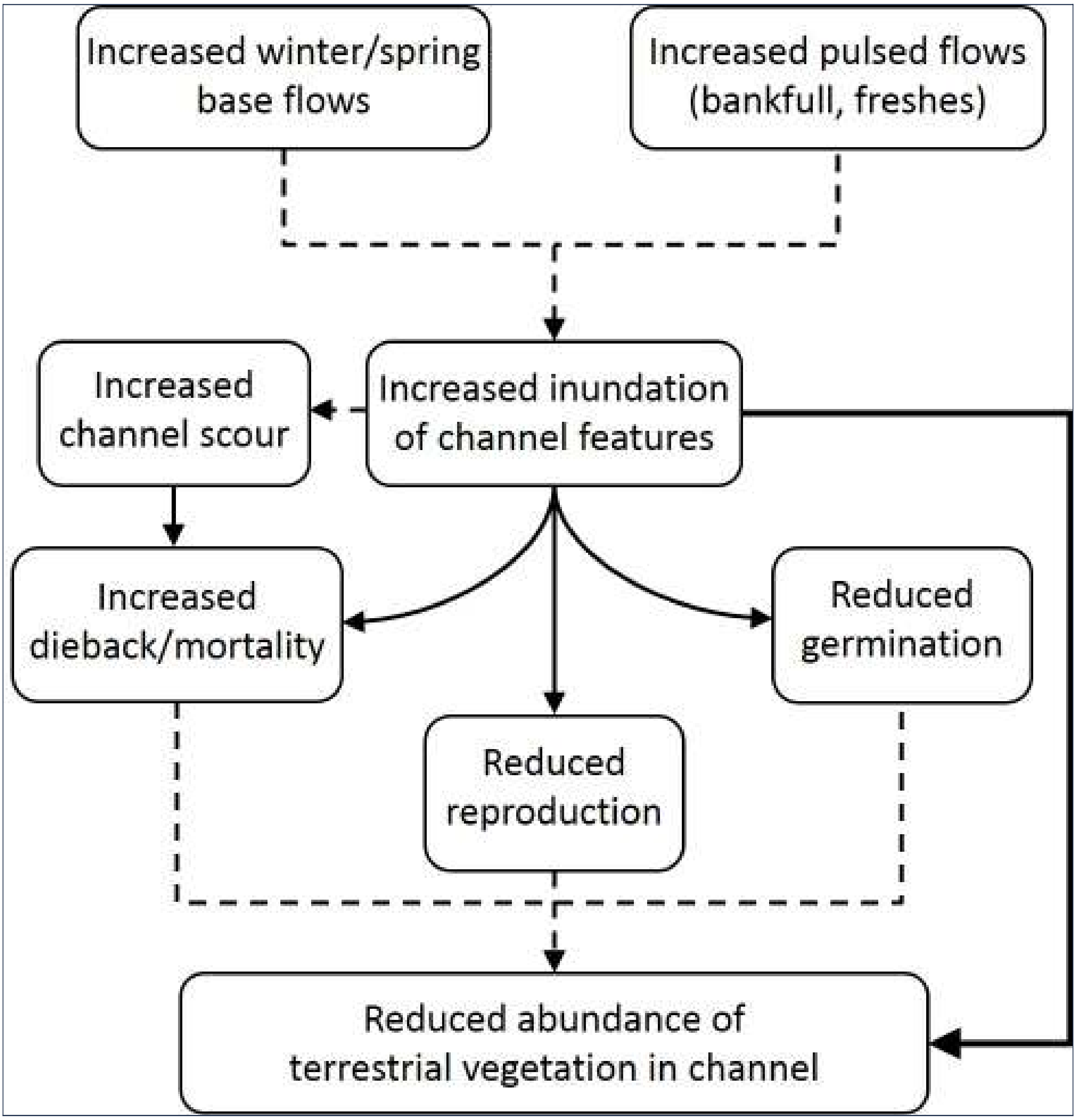

Literature review

Table 1: Results

The evidence according to the 3 major causal criteria shows whether the analysis provides enough support for a causal relationship between the hypothesised effect-cause linkages or alternatively whether there is no support, insufficient evidence or inconsistent evidence for the causal relationship. The minimum requirement for demonstration of a causal relationship is either "Response" or "Dose-response" to be HIGH, and also "Consistency" needs to be HIGH. Also shown are the number of studies and citations contributing to the analysis of each linkage.

Linkage	Conclusion regarding the level of support for the hypothesised linkage	Level of support for each criterion (sum of weights) *			Item counts		Number of studies reporting signs of causal agent in the biota
		Response	Dose-response	Consistency	Evidence items **	Citations	
↑ Inundation → ↓ vegetation (reproduction)	Insufficient evidence	Low (11)	No evidence (0)	High (10)	5	5	1
	Total number of evidence items and citations contributing to causal analysis				5	5	1

* Summed study weights for the different causal criteria. For "Response" and "Dose-response" criteria, if the summed study weight is less than 20 then the level of support is LOW, otherwise it is HIGH. For "Consistency" criteria, if the summed study weight is less than 20 then the level of support is HIGH, otherwise it is LOW.
** The number of relevant evidence items contributing to the analysis. Relevance is determined (and documented) by the user. For evidence to be included, the study must also conduct an appropriate analysis/interpretation. The project file contains the justification for including or excluding each evidence item.

Appendix

Table 2: Evidence relating to each cause-effect linkage

↑ Inundation → ↓ vegetation (reproduction)

Cause (and trajectory)		Effect (and trajectory)		Supports linkage?	Study details	Weight	Citation
Hydrocoytle umbellata seedlings, experimental treatments: increase in height of water from 0 cm to 15 cm to 30 cm, or flooded early, mid or late summer for around 35 days.	Increase	The only treatment in which Hydrocoytle umbellata plants flowered to any significant extent was the non-flooded treatment (Table 1). Non-flooded plants allocated about 20% of total mass to flower production at the final harvest. Although none of the plants had flowers at the final harvest in the mid- and late-summer treatments, plants in both of these treatments flowered prior to flooding earlier in the experiment, but all flowers aborted upon flooding (data not presented).	Decrease	Yes	Reference/control vs. impact (no before) 1 (control); 5 (impacted)	7	Dawe, C.E., Reekie, E.G. (2007)
Increase in frequency of inundation of vegetation near channels	Increase	Lower richness of native seed inputs (i.e,: seeds of fewer species set)	Decrease	Yes	Reference/control vs. impact (no before) 1 (control); 1 (impacted)	4	Tabacchi E., Planty-Tabacchi A. M., Roques L. and Nadal E. (2005)
Presence of flood water in experimental pools (i.e.: above "pool level" depth), to mimic an increase in volume of spring pulse and flood	Increase	Asexual reproduction by vegetative propagation of the exotic grass Phyla canescens during inundation.	Increase	No	Reference/control vs. impact (no before) 1 (control); 1 (impacted)	4	Taylor B. and Ganf G. G. (2005)
Large peak flood, ~1.78 times the mean annual flood for the century	Increase	Stem breakage during scour results in increased vegetative propagation from stem roots and nodes of an invasive perennial herb, Ageratina adenophora, in riparian areas.	Increase	No	Gradient response model 1 (independent)	3	Wang, R. Wang, J. F. Qiu, Z. J. Meng, B. Wan, F. H. Wang, Y. Z. (2011)
Peak river flows in spring, capable of uprooting trees.	Increase	Vegetative propagation of fragments and whole trees after scour events. 36-56% of uprooted Populus, Salix, and Alnus trees underwent vegetative propagation.	Increase	No	Gradient response model 1 (independent)	3	Francis, R. A. (2007)

Citations

Dawe, C.E., Reekie, E.G. (2007) *The effects of flooding regime on the rare Atlantic coastal plain species Hydrocoytle umbellata*. Canadian Journal of Botany-Revue Canadienne De Botanique

Francis, R. A. (2007) *Size and position matter: riparian plant establishment from fluvially deposited trees*. Earth Surface Processes and Landforms

Tabacchi E., Planty-Tabacchi A. M., Roques L. and Nadal E. (2005) *Seed inputs in riparian zones: Implications for plant invasion*. River Research and Applications

Taylor B. and Ganf G. G. (2005) *Comparative ecology of two co-occurring floodplain plants: the native Sporobolus mitchellii and the exotic Phyla canescens*. Marine and Freshwater Research

Wang, R. Wang, J. F. Qiu, Z. J. Meng, B. Wan, F. H. Wang, Y. Z. (2011) *Multiple mechanisms underlie rapid expansion of an invasive alien plant.*

New Phytologist

Table 3. Weights applied in this analysis

Study design type	Weight
BACI or BARI MBACI or Beyond MBACI	4
Gradient response model	3
Before v. after (no reference/control)	2
Reference/control vs. impact (no before)	2
After impact only	1
**Number of independent control locations**	**Weight**
No control locations	0
One control location	2
More than one control location	3
**Number of independent impact locations**	**Weight**
One impacted location	0
Two impacted locations	2
More than two impacted locations	3
**Number of locations for gradient response model**	**Weight**
3 independent locations	0
4 independent locations	2
5 independent locations	4
More than 5 independent locations	6
