# Supplementary material for: Environmental Flows Can Reduce the Encroachment of Terrestrial Vegetation into River Channels: A Systematic Literature Review
Source: Environ Manage. 2013 Aug 17;52(5):1202–12. doi: 10.1007/s00267-013-0147-0 (PMC3825610; doi:10.1007/s00267-013-0147-0)
Supplement: Supplementary file 4 — Supplementary material 4 (PDF 148 kb) [file 267_2013_147_MOESM4_ESM.pdf]

Eco Evidence: Analysis report

Problem

Environmental flows can reduce the encroachment of terrestrial vegetation into river channels: a systematic literature review

Question

An increase in inundation will cause a decrease in seed germination.

Context

Studies were considered relevant to our review if they presented primary data on the responses of terrestrial vegetation on lowland riverbanks or in channels, to changes in inundation regime. Studies from regulated and unregulated rivers, as well as comparable laboratory experiments were considered relevant. The vegetation response did not have to be the primary focus of the study; for example, the impacts of a scouring flood may have been described in a study comparing sites with differing levels of livestock access. The data could refer to either an increase or decrease in flows, and may be a result of natural variation in flow or anthropogenic streamflow alteration.

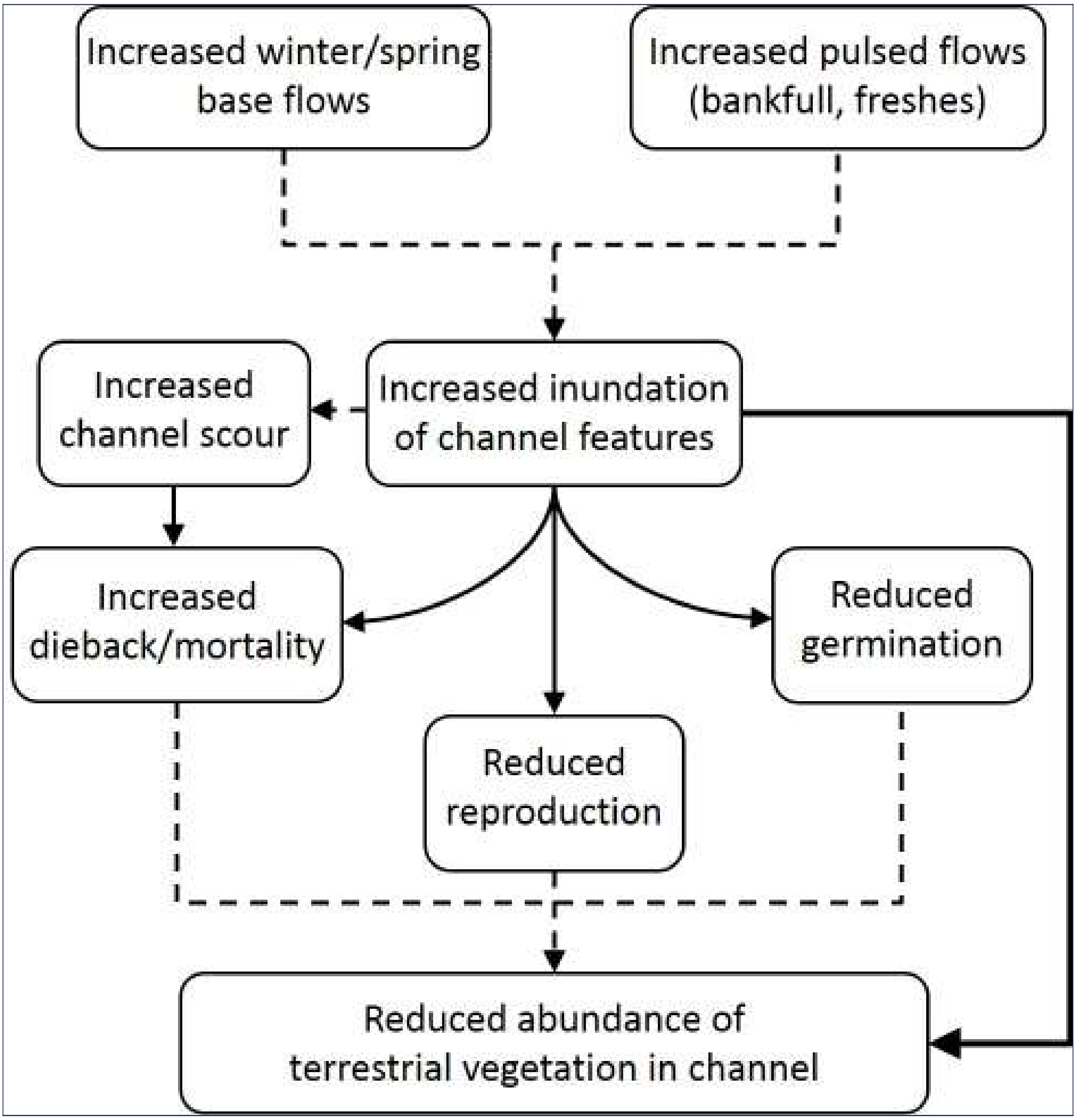

Literature review

Table 1: Results

The evidence according to the 3 major causal criteria shows whether the analysis provides enough support for a causal relationship between the hypothesised effect-cause linkages or alternatively whether there is no support, insufficient evidence or inconsistent evidence for the causal relationship. The minimum requirement for demonstration of a causal relationship is either "Response" or "Dose-response" to be HIGH, and also "Consistency" needs to be HIGH. Also shown are the number of studies and citations contributing to the analysis of each linkage.

Linkage	Conclusion regarding the level of support for the hypothesised linkage	Level of support for each criterion (sum of weights) *			Item counts		Number of studies reporting signs of causal agent in the biota
		Response	Dose-response	Consistency	Evidence items **	Citations	
↑ Inundation → ↓ vegetation (germination)	Support for alternate hypothesis	Low (10)	No evidence (0)	Low (24)	10	10	0
↓ Inundation → ↑ vegetation (germination)	Insufficient evidence	Low (3)	No evidence (0)	High (0)	1	1	0
	Total number of evidence items and citations contributing to causal analysis				11	11	0

* Summed study weights for the different causal criteria. For "Response" and "Dose-response" criteria, if the summed study weight is less than 20 then the level of support is LOW, otherwise it is HIGH. For "Consistency" criteria, if the summed study weight is less than 20 then the level of support is HIGH, otherwise it is LOW.
** The number of relevant evidence items contributing to the analysis. Relevance is determined (and documented) by the user. For evidence to be included, the study must also conduct an appropriate analysis/interpretation. The project file contains the justification for including or excluding each evidence item.

Appendix

Table 2: Evidence relating to each cause-effect linkage

↑ Inundation → ↓ vegetation (germination)

Cause (and trajectory)		Effect (and trajectory)		Supports linkage?	Study details	Weight	Citation
Inundation of reservoir margins	Increase	Greater germination from seed (by sexual reproduction, as opposed to asexual reproduction from clonal ramets) in more inundated sites.	Increase	No	Gradient response model<br>1 (independent)	3	Stokes, KE (2008)
Shifting of channel resulted in the presence of surface water during several months (Jul-Sept).	Increase	Presence of only mature (>7 years) individual Tamarix chinensis in flooded sites in its native habitat.	Decrease	Yes	Reference/control vs. impact (no before)<br>1 (control);<br>1 (impacted)	4	Cui, B. S. Yang, Q. C. Zhang, K. J. Zhao, X. S. You, Z. Y. (2010)
Cool-moist stratification (i.e.: inundation of seed capsules at 4 degrees C) for 61 days in experimental conditions.	Increase	Greater proportion of capsule germination 14-62 days after plants Gaura neomexicana spp coloradensis, a short-lived perennial forb.	Increase	No	Reference/control vs. impact (no before)<br>1 (control);<br>1 (impacted)	4	Burgess, L. M. Hild, A. L. Shaw, N. L. (2005)
Maintenance of near-bankfull flows after spring floods and throughout the growing season results in no stage recession.	Increase	Little to no recruitment of seedlings in the floodplain, which normally recruit during stage recession	Decrease	Yes	Gradient response model<br>1 (independent)	3	Braatne, J. H. Jamieson, R. Gill, K. M. Rood, S. B. (2007)
Zones of the channel and floodplain, mapped according to the discharge required in order to be inundated.	Increase	Higher densities of cottonwood (Populus deltoides, terrestrial damp species) seedlings on the floodplain zone, requiring higher volumes of water to inundate sites.	Increase	No	Gradient response model<br>1 (independent)	3	Auble G. T., Scott M. L., Friedman J. M., Back J. and Lee V. J. (1997)
22 months of flooding following a high rainfall event in a creek that had not flowed in the previous 100 years.	Increase	Large increase in tree germination (exotic Nicotiana glauca) in the 5 years following the initial flood	Increase	No	Reference/control vs. impact (no before)<br>1 (control);<br>1 (impacted)	4	Florentine S. K. and Westbrooke M. E. (2005)
Large winter peak floods and high flows in spring.	Increase	Establishment of Fremont cottonwoods	Increase	No	Gradient response model<br>1 (independent)	3	Stromberg J. (1998)
22 months of flooding following a high-rainfall event in a creek that had not flowed in the previous 100 years.	Increase	Nine species germinated only in flooded plots, not in non-flooded plots	Increase	No	Reference/control vs. impact (no before)<br>1 (control);<br>1 (impacted)	4	Westbrooke, M. E. Florentine, S. K. (2005)
Increase in the depth of surface water in experimental pools (drained, waterlogged, or submerged)	Increase	Lower germination success (propagules/m^2) from propagule banks.	Decrease	Yes	Gradient response model<br>3 (independent)	3	Gurnell A., Goodson J., Thompson K., Mountford O., and Clifford N. (2007)
Mean annual duration		Number of riparian vegetation	No		Gradient response		Pettit N. E., Froend

(number of days) of inundation	Increase	seedlings	change	No	model<br>2 (independent)	3	R. H. and Davies P. M. (2001)

↓ Inundation → ↑ vegetation (germination)

Cause (and trajectory)		Effect (and trajectory)		Supports linkage?	Study details	Weight	Citation
Low annual peak flows	Decrease	Germination of woody species (Populus and Tamarix) at lower elevations and in gravel bars and channels/islands.	Increase	Yes	Gradient response model<br>2 (independent)	3	Cooper D. J., Andersen D. C. and Chimner R. A. (2003)

Citations

Auble G. T., Scott M. L., Friedman J. M., Back J. and Lee V. J. (1997) *Constraints on establishment of plains cottonwood in an urban riparian preserve*. Wetlands

Braatne, J. H. Jamieson, R. Gill, K. M. Rood, S. B. (2007) *Instream flows and the decline of riparian cottonwoods along the Yakima River, Washington, USA*. River Research and Applications

Burgess, L. M. Hild, A. L. Shaw, N. L. (2005) *Capsule treatments to enhance seedling emergence of Gaura neomexicana ssp coloradensis*. Restoration Ecology

Cooper D. J., Andersen D. C. and Chimner R. A. (2003) *Multiple pathways for woody plant establishment on floodplains at local to regional scales*. Journal of Ecology

Cui, B. S. Yang, Q. C. Zhang, K. J. Zhao, X. S. You, Z. Y. (2010) *Responses of saltcedar (Tamarix chinensis) to water table depth and soil salinity in the Yellow River Delta, China*. Plant Ecology

Florentine S. K. and Westbrooke M. E. (2005) *Invasion of the noxious weed Nicotiana glauca R. Graham after an episodic flooding event in the arid zone of Australia*. Journal of Arid Environments

Gurnell A., Goodson J., Thompson K., Mountford O., and Clifford N. (2007) *Three seedling emergence methods in soil seed bank studies: implications for interpretation of propagule deposition in riparian zones*. Seed Science Research

Pettit N. E., Froend R. H. and Davies P. M. (2001) *Identifying the natural flow regime and the relationship with riparian vegetation for two contrasting western Australian rivers*. Regulated Rivers-Research & Management , 17 , 3 , 201-215

Stokes, KE (2008) *Exotic invasive black willow (Salix nigra) in Australia: influence of hydrological regimes on population dynamics*. PLANT ECOLOGY

Stromberg J. (1998) *Dynamics of Fremont cottonwood (Populus fremontii) and saltcedar (Tamarix chinensis) populations along the San Pedro River, Arizona*. Journal of Arid Environments

Westbrooke, M. E. Florentine, S. K. (2005) *Rainfall-driven Episodic Flood Events: are they a major factor in moulding New South Wales arid land vegetation patterns?*. Australian Geographer

Table 3. Weights applied in this analysis

Study design type	Weight
BACI or BARI MBACI or Beyond MBACI	4
Gradient response model	3
Before v. after (no reference/control)	2
Reference/control vs. impact (no before)	2
After impact only	1
**Number of independent control locations**	**Weight**
No control locations	0
One control location	2
More than one control location	3
**Number of independent impact locations**	**Weight**
One impacted location	0
Two impacted locations	2
More than two impacted locations	3
**Number of locations for gradient response model**	**Weight**
3 independent locations	0
4 independent locations	2
5 independent locations	4
More than 5 independent locations	6
