# Supplementary material for: Environmental Flows Can Reduce the Encroachment of Terrestrial Vegetation into River Channels: A Systematic Literature Review
Source: Environ Manage. 2013 Aug 17;52(5):1202–12. doi: 10.1007/s00267-013-0147-0 (PMC3825610; doi:10.1007/s00267-013-0147-0)
Supplement: Supplementary file 5 — Supplementary material 5 (PDF 159 kb) [file 267_2013_147_MOESM5_ESM.pdf]

Eco Evidence: Analysis report

Problem

Environmental flows can reduce the encroachment of terrestrial vegetation into river channels: a systematic literature review

Question

An increase in inundation will lead to a decrease in abundance.

Context

Studies were considered relevant to our review if they presented primary data on the responses of terrestrial vegetation on lowland riverbanks or in channels, to changes in inundation regime. Studies from regulated and unregulated rivers, as well as comparable laboratory experiments were considered relevant. The vegetation response did not have to be the primary focus of the study; for example, the impacts of a scouring flood may have been described in a study comparing sites with differing levels of livestock access. The data could refer to either an increase or decrease in flows, and may be a result of natural variation in flow or anthropogenic streamflow alteration.

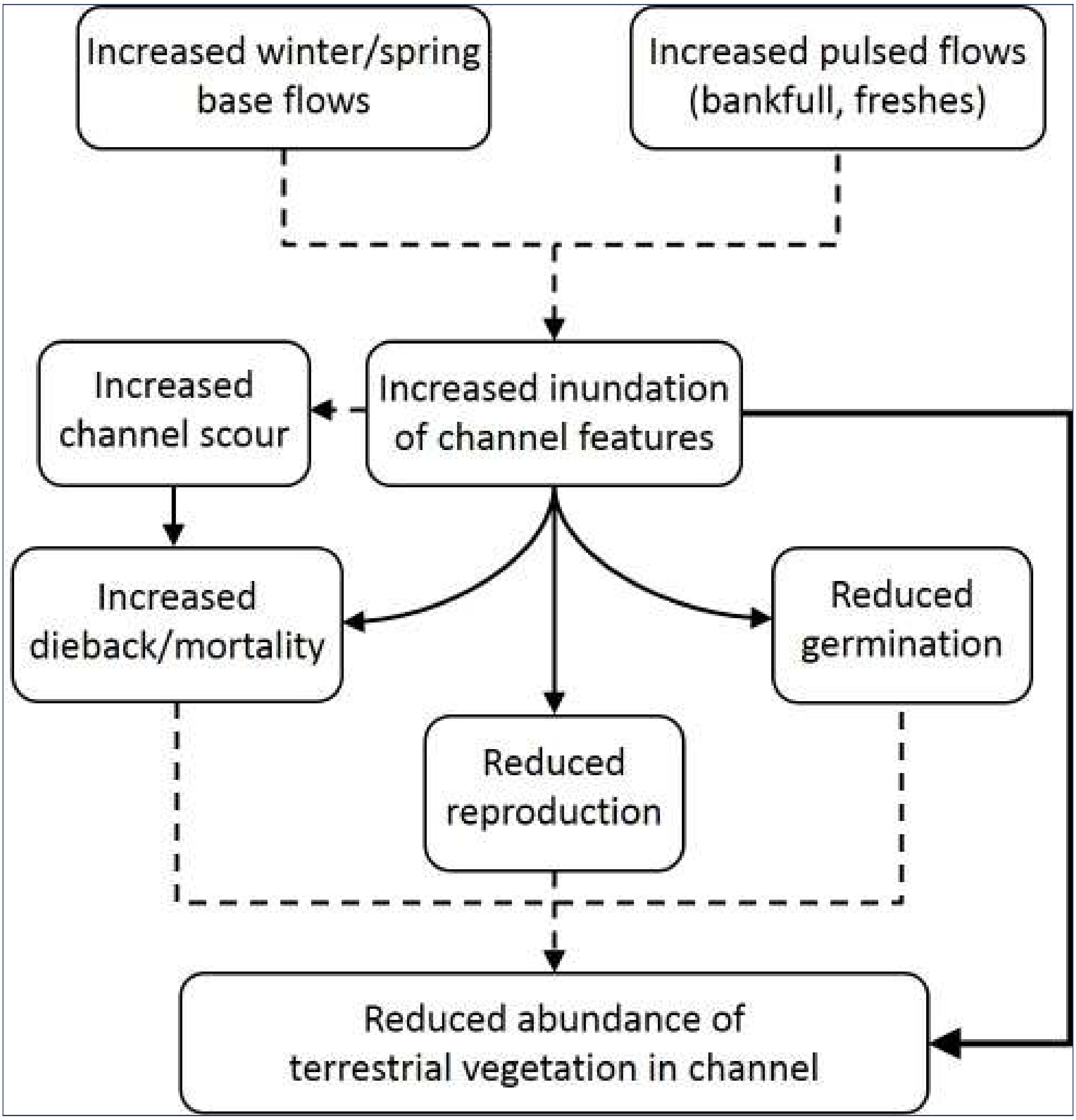

Literature review

Table 1: Results

The evidence according to the 3 major causal criteria shows whether the analysis provides enough support for a causal relationship between the hypothesised effect-cause linkages or alternatively whether there is no support, insufficient evidence or inconsistent evidence for the causal relationship. The minimum requirement for demonstration of a causal relationship is either "Response" or "Dose-response" to be HIGH, and also "Consistency" needs to be HIGH. Also shown are the number of studies and citations contributing to the analysis of each linkage.

Linkage	Conclusion regarding the level of support for the hypothesised linkage	Level of support for each criterion (sum of weights) *			Item counts		Number of studies reporting signs of causal agent in the biota
		Response	Dose-response	Consistency	Evidence items **	Citations	
↑ Inundation → ↓ vegetation (abundance)	Support for hypothesis	High (50)	No evidence (0)	High (10)	16	16	1
↓ Inundation → ↑ vegetation (abundance)	Insufficient evidence	Low (10)	No evidence (0)	High (3)	4	4	0
	Total number of evidence items and citations contributing to causal analysis				20	20	1

* Summed study weights for the different causal criteria. For "Response" and "Dose-response" criteria, if the summed study weight is less than 20 then the level of support is LOW, otherwise it is HIGH. For "Consistency" criteria, if the summed study weight is less than 20 then the level of support is HIGH, otherwise it is LOW.
** The number of relevant evidence items contributing to the analysis. Relevance is determined (and documented) by the user. For evidence to be included, the study must also conduct an appropriate analysis/interpretation. The project file contains the justification for including or excluding each evidence item.

Appendix

Table 2: Evidence relating to each cause-effect linkage

↑ Inundation → ↓ vegetation (abundance)

Cause (and trajectory)		Effect (and trajectory)		Supports linkage?	Study details	Weight	Citation
Mean annual number of flooding events (i.e.: flooding in sampled plots)	Increase	Decline in % cover of shrubs	Decrease	Yes	Gradient response model 2 (independent)	3	Pettit N. E., Froend R. H. and Davies P. M. (2001)
Frequency of inundation by peak floods: annual, 3-5y, and 100y	Increase	Lower site occupancy and stem density of Populus and Tamarix on floodplains with the highest frequencies of flooding. Salix had similar site occupancy at annually and 3-5y flooding sites, but densities were lower at annually flooding sites for all age classes	Decrease	Yes	Gradient response model 1 (independent)	3	Irvine, J.R. West, N.E. (1979)
Number of years since last disturbance, defined as the presence of the stream channel within a plot.	Increase	No significant relationship between number of years passed and percent ground over of woody species. Very weak, but statistically significant, relationship for herbaceous species.	No change	No	After impact only 0 (control); 1 (impacted)	1	Tiegs S. D., O'Leary J. F., Pohl M. M. and Munill C. L. (2005)
Percentage of recorded high tide events (28/month) where marshes were flooded	Increase	Stem density of Phragmites australis in wetlands	Increase	No	Gradient response model 1 (independent)	3	Chambers, R. M. Osgood, D. T. Kalapasev, N. (2002)
Inundation depth	Increase	Percent cover of herbaceous vegetation	Increase	No	Gradient response model 1 (independent)	3	Shafroth, P. B. Auble, G. T. Stromberg, J. C. Patten, D. T. (1998)
Duration of flood inundation following a high rainfall event in a creek that had not flowed in the previous 100 years.	Increase	Fewer trees (exotic Nicotiana glauca) in long-term flood zones than short-term flood zones. Nonflooded zones, however, had the fewest trees [not included in this evidence item].	Decrease	Yes	Gradient response model 1 (independent)	3	Florentine S. K. and Westbrooke M. E. (2005)
Greater depth of surface water in temporary-flooded wetlands	Increase	Lower proportional cover of terrestrial species (natives and exotics)	Decrease	Yes	Gradient response model 1 (independent)	3	Catford, J. A. Downes, B. J. (2010)
Restoration of river by dechannelization and ditch-filling lead to increased water depths in relict marshes on the floodplain.	Increase	Decrease in cover of terrestrial/non-wetland species measured by mean derived cover. This decreased abundance (near total elimination) results in an overall decrease in species richness [not included in this evidence item].	Decrease	Yes	Gradient response model 2 (independent)	3	Toth, L. A. (2010)
Higher water levels during spring (Feb-May); ~115-120		Decrease in percent cover of			Before v. after (no		Jenkins, N. J.,

cm higher than previous years. Increased water depth is confounded by a longer duration of flooding.	Increase	invasive reed canary grass (Phalaris arundinacea), particularly where inundation was >0.85 m.	Decrease	Yes	reference/control) 0 (control); 1 (impacted)	2	Yeakley, J. A. and Stewart, E. M. (2008)
Increase in depth of experimental pools to mimic an increase in volume of spring pulse and flood	Increase	Suppression of proliferation (% cover) of Sporobolus mitchelli (native grass) and Phyla canescens (exotic) while inundated. However, after flood recession, plant growth recommenced.	Decrease	Yes	Reference/control vs. impact (no before) 1 (control); 1 (impacted)	4	Taylor B. and Ganf G. G. (2005)
Dechannelization led to an increase in mean annual hydroperiod (number of days of inundation) from 96+/-8 to 347+/-6 in the floodplain	Increase	Near elimination of mesophytic shrub and fern cover on the floodplain	Decrease	Yes	BACI or BARI MBACI or Beyond MBACI 1 (control); 1 (impacted)	6	Toth, L. A. (2010)
Large peak flood, ~1.78 times the mean annual flood for the century	Increase	High density of invasive perennial herb perennial herb, Ageratina adenophora, in riparian areas previously damaged by large flood, which can reproduce by vegetative propagation.	Increase	No	Gradient response model 1 (independent)	3	Wang, R. Wang, J. F. Qiu, Z. J. Meng, B. Wan, F. H. Wang, Y. Z. (2011)
Permanence of streamflow (intermittent or perennial)	Increase	Lower patch cover of exotic Tamarix in floodplains of intermittent reaches than perennial reaches. NB: Authors detected the opposite pattern for native floodplain veg (Populus and Salix).	Decrease	Yes	Reference/control vs. impact (no before) 5 (control); 5 (impacted)	8	Stromberg, J. C. Lite, S. J. Marler, R. Paradzick, C. Shafroth, P. B. Shorrock, D. White, J. M. White, M. S. (2007)
Longer duration of summer flooding	Increase	Restriction of the lower distribution of 10 grassland species, with more flood-sensitive species more restricted. NB: Authors id not detect a relationship between winder flood duration and species distribution.	Decrease	Yes	Gradient response model 1 (independent)	3	van Eck, Whjm Lenssen, J. P. M. van de Steeg, H. M. Blom, Cwpm de Kroon, H. (2006)
Duration of complete inundation in experimental treatments.	Increase	Total plant biomass was reduced with longer durations of inundation in 20 terrestrial plant species.	Decrease	Yes	Gradient response model 20 (independent)	9	van Eck, Whjm van de Steeg, H. M. Blom, Cwpm de Kroon, H. (2004)
Increasing stream depth and/or distance from the bank	Increase	Occurrence of terrestrial plants in transects of 29 stream systems.	Decrease	Yes	Gradient response model 0 (independent)	3	Riis, T. Sand-Jensen, K. Larsen, S. E. (2001)

↓ Inundation → ↑ vegetation (abundance)

Cause (and trajectory)		Effect (and trajectory)		Supports linkage?	Study details	Weight	Citation
Percentage of days in the 20-year period preceding sampling in which flow exceeded commence-to-fill values. (i.e.: the proportion of days where sites were flooded in the previous 20 years)	Decrease	Mean species cover values of understorey vegetation in grass plain wetland sites.	No change	No	Gradient response model 1 (independent)	3	Stokes, K. Ward, K. Colloff, M. (2010)
Reduction in maximum flood depth due to river regulation	Decrease	Increased abundance of terrestrial exotic weeds in riparian wetlands, as a proportion of total vegetation cover. Proportional cover of terrestrial native species (as a group) did not relate to hydrological modification, but increased cover of individuals species did relate to decreased water depth.	Increase	Yes	Gradient response model 1 (independent)	3	Catford, J. A. Downes, B. J. Gippel, C. J. Vesk, P. A. (2011)
Presence of surface water during several months (Jul-Sept)	Decrease	Density (Individuals/100 m^2) of Tamarix chinensis in its native habitat	Increase	Yes	Reference/control vs. impact (no before) 1 (control); 1 (impacted)	4	Cui, B. S. Yang, Q. C. Zhang, K. J. Zhao, X. S. You, Z. Y. (2010)
Decline in open-water area by 49% during the restoration of a shallow, water-emergent system in a wetland, after the decline in lake levels of nearby Lake Erie.	Decrease	Increase in the absolute and proportional cover of emergent vegetation, including Phragmites, Typha, Sparganium, and Sagittaria, relative to macrophytes.	Increase	Yes	Gradient response model 1 (independent)	3	Whyte, R. S. Trexel-Kroll, D. Klarer, D. M. Shields, R. Francko, D. A. (2008)

Citations

Catford, J. A. Downes, B. J. (2010) *Using multi-scale species distribution data to infer drivers of biological invasion in riparian wetlands.* Diversity and Distributions

Catford, J. A. Downes, B. J. Gippel, C. J. Vesk, P. A. (2011) *Flow regulation reduces native plant cover and facilitates exotic invasion in riparian wetlands.* Journal of Applied Ecology

Chambers, R. M. Osgood, D. T. Kalapasev, N. (2002) *Hydrologic and chemical control of Phragmites growth in tidal marshes of SW Connecticut, USA.* Marine Ecology-Progress Series

Cui, B. S. Yang, Q. C. Zhang, K. J. Zhao, X. S. You, Z. Y. (2010) *Responses of saltcedar (Tamarix chinensis) to water table depth and soil salinity in the Yellow River Delta, China*. Plant Ecology

Florentine S. K. and Westbrooke M. E. (2005) *Invasion of the noxious weed Nicotiana glauca R. Graham after an episodic flooding event in the arid zone of Australia*. Journal of Arid Environments

Irvine, J.R. West, N.E. (1979) *Riparian Tree Species Distribution and Succession along the Lower Escalante River, Utah*. The Southwestern Naturalist

Jenkins, N. J., Yeakley, J. A. and Stewart, E. M. (2008) *First-year responses to managed flooding of Lower Columbia River bottomland vegetation dominated by Phalaris arundinacea*. Wetlands

Pettit N. E., Froend R. H. and Davies P. M. (2001) *Identifying the natural flow regime and the relationship with riparian vegetation for two contrasting western Australian rivers*. Regulated Rivers-Research & Management

Riis, T. Sand-Jensen, K. Larsen, S. E. (2001) *Plant distribution and abundance in relation to physical conditions and location within Danish stream systems*. Hydrobiologia

Shafroth, P. B. Auble, G. T. Stromberg, J. C. Patten, D. T. (1998) *Establishment of woody riparian vegetation in relation to annual patterns of streamflow, Bill Williams River, Arizona*. Wetlands

Stokes, K. Ward, K. Colloff, M. (2010) *Alterations in flood frequency increase exotic and native species richness of understorey vegetation in a temperate floodplain eucalypt forest*. Plant Ecology , 211 , 2 , 219-233

Stromberg, J. C. Lite, S. J. Marler, R. Paradzick, C. Shafroth, P. B. Shorrock, D. White, J. M. White, M. S. (2007) *Altered stream-flow regimes and invasive plant species: the Tamarix case*. Global Ecology and Biogeography

Taylor B. and Ganf G. G. (2005) *Comparative ecology of two co-occurring floodplain plants: the native Sporobolus mitchellii and the exotic Phyla canescens*. Marine and Freshwater Research

Tiegs S. D., O'Leary J. F., Pohl M. M. and Munill C. L. (2005) *Flood disturbance and riparian species diversity on the Colorado River Delta*. Biodiversity and Conservation

Toth, L. A. (2010) *Restoration Response of Relict Broadleaf Marshes to Increased Water Depths*. Wetlands

Toth, L. A. (2010) *Unrealized Expectations for Restoration of a Floodplain Plant Community*. Restoration Ecology

van Eck, Whjm Lenssen, J. P. M. van de Steeg, H. M. Blom, Cwpm de Kroon, H. (2006) *Seasonal dependent effects of flooding on plant species survival and zonation: a comparative study of 10 terrestrial grassland species*. Hydrobiologia

van Eck, Whjm van de Steeg, H. M. Blom, Cwpm de Kroon, H. (2004) *Is tolerance to summer flooding correlated with distribution patterns in river floodplains? A comparative study of 20 terrestrial grassland species*. Oikos

Wang, R. Wang, J. F. Qiu, Z. J. Meng, B. Wan, F. H. Wang, Y. Z. (2011) *Multiple mechanisms underlie rapid expansion of an invasive alien plant*. New Phytologist

Whyte, R. S. Trexel-Kroll, D. Klarer, D. M. Shields, R. Francko, D. A. (2008) *The Invasion and Spread of Phragmites australis during a Period of Low Water in a Lake Erie Coastal Wetland.* Journal of Coastal Research

Table 3. Weights applied in this analysis

Study design type	Weight
BACI or BARI MBACI or Beyond MBACI	4
Gradient response model	3
Before v. after (no reference/control)	2
Reference/control vs. impact (no before)	2
After impact only	1
**Number of independent control locations**	**Weight**
No control locations	0
One control location	2
More than one control location	3
**Number of independent impact locations**	**Weight**
One impacted location	0
Two impacted locations	2
More than two impacted locations	3
**Number of locations for gradient response model**	**Weight**
3 independent locations	0
4 independent locations	2
5 independent locations	4
More than 5 independent locations	6
